# Opportunities and Possibilities of Developing an Advanced Precision Spraying System for Tree Fruits

**DOI:** 10.3390/s21093262

**Published:** 2021-05-08

**Authors:** Md Sultan Mahmud, Azlan Zahid, Long He, Phillip Martin

**Affiliations:** 1 Department of Agricultural and Biological Engineering, The Pennsylvania State University, University Park, PA 16802, USA; mvm6735@psu.edu (M.S.M.); axz264@psu.edu (A.Z.); 2 Fruit Research and Extension Center, The Pennsylvania State University, Biglerville, PA 17307, USA; plm30@psu.edu; 3 Department of Plant Pathology and Environmental Microbiology, The Pennsylvania State University, University Park, PA 16803, USA

**Keywords:** crop protection, canopy detection, canopy density, canopy volume, deep learning, machine vision, sensing

## Abstract

Reducing risk from pesticide applications has been gaining serious attention in the last few decades due to the significant damage to human health, environment, and ecosystems. Pesticide applications are an essential part of current agriculture, enhancing cultivated crop productivity and quality and preventing losses of up to 45% of the world food supply. However, inappropriate and excessive use of pesticides is a major rising concern. Precision spraying addresses these concerns by precisely and efficiently applying pesticides to the target area and substantially reducing pesticide usage while maintaining efficacy at preventing crop losses. This review provides a systematic summary of current technologies used for precision spraying in tree fruits and highlights their potential, briefly discusses factors affecting spraying parameters, and concludes with possible solutions to reduce excessive agrochemical uses. We conclude there is a critical need for appropriate sensing techniques that can accurately detect the target. In addition, air jet velocity, travel speed, wind speed and direction, droplet size, and canopy characteristics need to be considered for successful droplet deposition by the spraying system. Assessment of terrain is important when field elevation has significant variability. Control of airflow during spraying is another important parameter that needs to be considered. Incorporation of these variables in precision spraying systems will optimize spray decisions and help reduce excessive agrochemical applications.

## 1. Introduction

Tree fruits are high-value commodities worth almost $18 billion in the U.S. [1]. Tree fruits require pesticides for consistent production, because failure to control insects, pests, disease, and weeds results in losses of up to 100% of the crop [2,3]. However, pesticide use in orchards has come under scrutiny for contaminating the air, soil, and sub-surface water, which is considered hazardous to the health and wellbeing of people, animals, and the environment [4,5,6,7]. Pesticides are also expensive, costing tree fruit growers hundreds to low thousands of dollars per hectare, which accounts for around a third of total production costs [8,9,10]. Pimentel and Burgess [11] showed that pesticide applications caused around $8.2 billion annual environmental and economic losses in the U.S. Currently, the conventional constant-rate air blast sprayers widely used in commercial-scale orchards are estimated to deposit less than 30% of the pesticides on target trees [12]. Most of the pesticide waste is from applications to the spaces above, below, and between tree canopies. Substantial reductions in pesticide waste and misuse can be achieved by precise and targeted applications of pesticides to the tree canopies.

Precision spraying is defined as the precise spraying of pesticides based on target information such as the size, shape, structure, and density of the tree canopy as obtained from sensors such as cameras, ultrasound, and laser. Using precision spraying technologies, Esau et al. [13] reduced agrochemical usages from 9.90% to 51.22% by not applying pesticides to bare spot areas in wild blueberry fields. Jejčič et al. [14] saved about 20.2% of spray volume by applying pesticides based on the characteristics of the targets in orchards. Asaei et al. [15] considered the spacing between trees along orchard rows for pesticide application and obtained a reduction of up to 54% of pesticide usage. Although several studies have reported a significant amount of pesticide reduction during spraying, most of them evaluated their savings on a small scale or in fairly controlled environments that are not feasible to a large-scale orchard field. Additionally, despite the advanced spraying technologies available in the past few years, there are still concerns with the amount of chemicals used and lost to drift [16], requiring more advanced technology for spraying in the large scale orchards. Precision sprayers are commercially available for weed control and plant treatment in arable land. However, very few precision spraying systems are commercially available for tree fruit orchards, and most of them are not fully compatible with successful spray deposition in real-time orchard conditions. Previously available reviews on precision spraying have focused mainly on plant canopy characterizations and flow rate calculations [17], sensing technologies used for field crops [18], and the empirical dose expression models applied for spray decision making [19].

This review focuses on the importance of advanced spraying systems, limitations of current technologies, factors affecting sprayer performance, and gaps in knowledge that need to be considered in the development of advanced precision sprayer systems for tree fruit orchards. Firstly, the history and revolution of sprayers and the limitations of current spraying methods were discussed. Then, the core components and technologies for precision spraying were identified and discussed, which are centered around camera, ultrasonic, and laser-sensing technologies. The factors effecting the use of these core sensing technologies were followed, including nozzle types, spraying distance, droplet size, canopy characteristics, wind speed, temperature, and relative humidity. Finally, possible pathways to address some of the challenges were discussed and concluded.

## 2. Overview of Sprayers and Spraying Systems

### 2.1. Chemical Sprayers in Tree Fruit Orchards

Over the past decades, orchard sprayer designs have evolved from hand-boom based horse-drawn wagons to sensor-controlled tractor-pulled sprayers, due to increased concern about chemical wastage and environmental contamination, changes in horticulture, increased demand for quality products, and development of new technologies. The revolution of sprayer design is listed in (Table 1). Various early devices for pesticide application were introduced back in the early 20th century, including pumps, nozzles, bellows, and steam-powered sprayers [20]. Given the labor shortage problems, early rapid adoption of air blast sprayers occurred in the 1940s. The strong farmer interest in this innovation encouraged a surge in the use of air-assisted spraying [21]. Boom sprayers and other innovations (e.g., mist blowers, handguns) from Europe were developed that allowed growers to apply pesticides more efficiently [22,23,24]. A significant change of chemical application strategies took place when the air jet models were developed that could carry the spray up and into the canopy [25]. The air jet models allowed chemicals to be sprayed from the ground instead of only being sprayed from planes. In the last two–three decades, modern sprayers including tunnel sprayers, tower sprayers, and precision sprayers have allowed orchard growers to reduce off-target deposition of spray materials and drift. Of these, precision or intelligent sprayers equipped with sensing technology have shown great potential to reduce excessive chemical applications [16,26].

**Figure 1:**
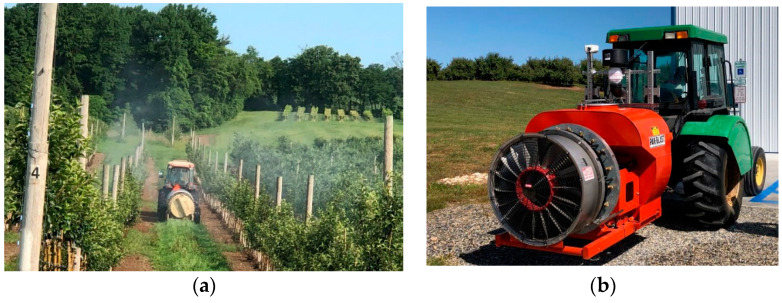
Air blast spraying systems (**a**) constant rate air blast sprayer; (**b**) intelligent sprayer.

Air blast sprayers (Figure 1) have been common in tree fruit orchards for a long time. The sprayers consist of a single fan located at the rear of the machine that pulls air in and redistributes it upwards into the tree canopy. The direction and volume of air are critical factors because the volume of air released from the fan must be matched with the tree canopy for appropriate spray deposition and coverage. These sprayers were introduced to orchards when trees were grown in the iconic architecture with a height greater than 6 m with many wide-spanning branches, and were planted in rows with spacings of 4–6 m. Fox et al. [32] showed that air blast sprayers were suitable for trees with such iconic architecture due to the large volume of air needed to push spray droplets 9 m into the tree canopy. However, many apple trees planted in the past few years are only 2 to 4 m tall and have smaller canopies, which creates problems in matching them to the air volume from conventional air blast sprayers [33]. Conventional sprayers bring significant challenges to matching the air output during spraying with what is needed for successful spray droplet deposition.

Air blast sprayers include conventional axial-fan air blast sprayers, cannon sprayers, tunnel sprayers, tower sprayers, and custom-designed or modified air blast sprayers. The axial fan air-assisted sprayer produces a large radial spray volume and is the predominant design of sprayer used in tree fruit orchards. It is poorly targeted for modern intensive tree fruit orchards and creates a significant risk of off-target contamination by spray drift and chemical wastage to the ground [33]. Tunnel and crossflow sprayers have been poorly utilized due to increased expenses and decreased operational adaptability [26]. There are also problems associated with spraying with tunnel sprayers because of diseases that might be spread by physical contact or by reused spray solution while driving such a large machine through the orchards [32]. Tower type sprayers, which direct the airflow from the fan into horizontal ducts on a vertical plane, have improved spray coverage in the top center of trees and work well for trees with consistent heights. Cannon sprayers are commonly small in size and only require a small path for spray application, but may not produce uniform spray deposition and coverage because their performance is influenced by wind speed.

### 2.2. Challenges with Conventional Sprayers

Conventional air blast sprayers designed for larger trees can waste more than 50% of pesticides to the ground and the atmosphere when spraying modern apple trees that have smaller canopies [34,35,36]. Spray applications in tree fruit orchards have been based on the application rate per unit area which was calculated from the number of rows and the average tree spacing. This was known as broadcast spraying, and did not automatically adjust for weather conditions, tree canopy density, missing trees, or the height and shape of the trees, and resulted in up to 60–70% off-target losses [37]. Precision spraying can substantially reduce off-target losses by using real-time tree canopy data to adjust the sprayers for precise and targeted pesticide applications.

## 3. Core Components and Technologies for Precision Spraying

### 3.1. Tree Canopy Parameter Measurements

With the advancement of modern sensors and technologies, the methods utilized for measuring complex tree canopy parameters are developing rapidly. These methods aim to accurately measure geometric canopy features, canopy density, canopy leaf area density (LAD), and canopy leaf area index (LAI) (Table 2). These measurements provide precise information to assess the accurate spray volume needed for specific trees.

**Geometric Canopy Dimensions:** Tree geometric canopy dimensions, such as canopy height, width, and volume, are needed for calculating the canopy density, leaf area density, and canopy leaf area index. Camera sensors are mainly used to detect the LWA (leaf wall area) and have limited use in detecting canopy volume. The main sensors used for canopy geometric characteristics measurements are ultrasonic and LiDAR. Beyaz and Dagtekin [44] measured citrus tree canopy volume using ultrasonic and camera imaging sensors and concluded that the ultrasonic sensor can achieve 15% better correlation compared to the imaging technique. However, the performance of ultrasonic sensors is influenced by the movement of the carrying medium. LiDAR is a special application of laser sensors that shows great promise in detecting canopy dimensions. Data from LiDAR are processed mostly with slice and cuboid algorithms for canopy volume measurement. Slice algorithms split the tree canopy horizontally or longitudinally while cuboid algorithms divide the tree canopy into different sized cuboids. Lee and Ehsani [45] used LiDAR sensor and slice algorithm to measure the citrus tree canopy geometric characteristics. The tree was split into several layers or slices along the detection path, and each slice was considered a symmetric polyhedron. The study reported the relative errors were −0.37%, 0.01%, −1.99%, and 5.96% for tree canopy height, width, surface area, and volume measurements, respectively. Yu et al. [46] also used the slice algorithm to calculate tree canopy volume through the LiDAR sensor and achieved a relative error of 5% compared with manual measurement. The authors concluded that the slice algorithm is mainly suitable for trees with symmetrical structures, and the error would be increased while tested with other structured trees. Mahmud et al. [47] measured tree canopy volume based on the cuboids method using a LiDAR sensor and alpha shape algorithm. The main advantage of using alpha shape algorithm is it allows for concave shapes that have a lower chance of overestimation. Using the alpha value of 1, this study achieved a very high correlation of 0.98 (R^2^ = 0.95). Cheraïet et al. [48] used a 2D LiDAR sensor for estimating canopy height and width in vineyards. A cuboid-based Bayesian point cloud classification algorithm (BPCC) was used to process LiDAR data, which combined an automatic filtering method (AFM) and a classification method based on clustering. Estimation of canopy height and width obtained strong correlations (R^2^ = 0.94 and 0.89, respectively) showed the potential of using this technique to adjust the spray rate for variable-rate spraying. Chen et al. [49] measured tree canopy characteristics as a plurality of cuboids along the longitudinal direction using LiDAR sensor. The study concludes this method can be useful for various tree canopy parameter measurements and the accuracy of measurement depends on the number of cuboids.

**Figure 2:**
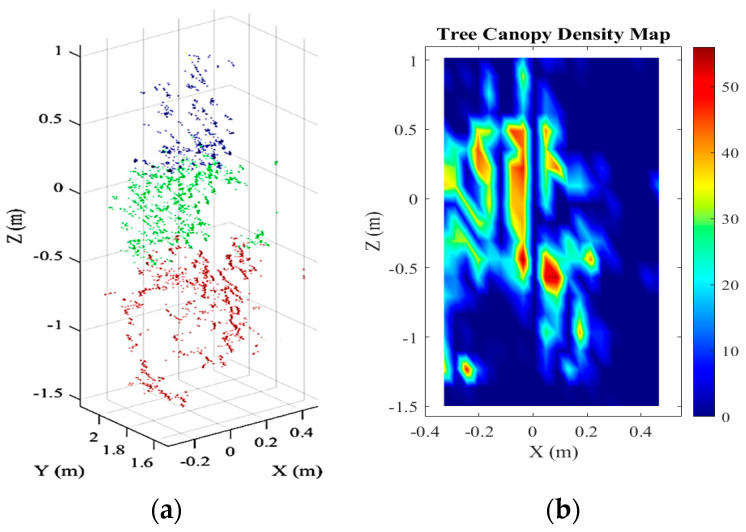
Canopy foliage density map (**a**) Tree canopy points without the trunk, trellis wires, and support pole points divided into sections; (**b**) canopy density map considering the number of leaves (per grid area) [47] (used with permission).

**Canopy Density:** Tree canopy density is essential to determine the spray volume for accurate variable-rate spray applications [47,50]. The canopy density is measured by dividing the number of leaves of a tree by the total leaf canopy area. Li et al. [51] utilized an ultrasonic-sensing method to build the relationship between canopy density and leaf layers using an orthogonal regression rotation test. The maximum relative errors were 29.92%, 21.33%, 25.64%, and 17.68% between the model values and the actual values. There was a close relationship between echo energy, detection distance, and canopy density. The study was conducted in regular/uniform leaf distribution, and this method is not practical to accurately calculate the density of canopies with non-uniform and irregular leaf positions. Hu and Whitty [50] used 2D LiDAR, three RGB-D cameras, and a GPS-RTK module to calculate apple tree canopy density and achieved an average co-efficient of determination (R^2^) of 0.90. Mahmud et al. [47] developed a section-based canopy density measurement system comprised of a Velodyne sick 3D light detection and ranging (LiDAR) sensor with series of processing algorithms. The density variability was measured within four tree sections divided by trellis wire positions (Figure 2). The study predicted the number of leaves in four sections using the developed models. The average percent errors of 24.44% and 14.21% were calculated for predicting the number of leaves in GoldRush and Fuji apple trees, respectively. The canopy density maps were generated with the predicted numbers of leaves for precision spraying. 

**Canopy Leaf Area Density:** Canopy leaf area density (LAD) is crucial for characterizing the differences in leaf area at different layers in the vertical direction and employing variable-rate control of nozzles [52]. The LAD is the unilateral leaf area of the photosynthetic tissue per unit canopy volume. The measurement of LAD requires multi-point positioning, which is best obtained with LiDAR sensors. The calculation of LAD is sensitive to volume calculation models, such as voxel models which require an accurate choice of voxel size. Wang et al. [52] calculated the LAD by segmenting the LiDAR point cloud from tree canopies and volume element models. A Voxel-based canopy profiling (VCP) method was used for LAD calculation and reported a relative error of 1.26%. Chakraborty et al. [53] compared the voxel grid and convex hull methods to measure canopy volume for apple trees and grapevines using 3D LiDAR. They reported the voxel gird method performed poorly with a correlation of 0.51 compared to the convex hull method of 0.81. The authors noted that the voxel gird method is affected by voxel size and this method is computationally intense. Béland et al. [54] mapped forest leaf area density through a radiative transfer simulation model, voxel method, and a multi-view terrestrial LiDAR. The voxel arrays were inserted into the radiative transfer model to simulate bidirectional reflectance factors and the vertical fraction of absorbed radiation. This study’s findings established initial guidelines for mapping leaf area density in dense tree areas. Berk et al. [55] reconstructed the tree canopy for calculating LAD through LiDAR sensor and the volume element method. The trapezoidal method was used to calculate individual volumes of tree canopy volume elements. The maximum correlation of 0.80 was achieved when establishing the relationship between tree canopy volume and LAD. The study concluded that the LAD’s accuracy depends on how accurately the size of volume element is estimated.

**Canopy Leaf Area Index:** The canopy leaf area index (LAI) is a core parameter of vegetation structure used to represent the leaf area per unit surface area [56]. It is a dimensionless quantity that can provide sufficient information for pesticide dose calculation for precise control sprayer nozzles. The LAI can be measured either directly or indirectly; the direct approach is time-consuming while the indirect method requires sensor applications. Arnó et al. [57] measured LAI using vehicle-mounted LiDAR sensors though scanning the tree. The tree area index achieved a strong R^2^ of 0.92; however, the insufficient number of scans and the complexity of the data analysis for LAI estimation were considered the major limitations of this study. Comba et al. [58] measured LAI from 3D point clouds acquired in vineyards using multispectral imagery and calculated through a multivariate linear regression model. Image data were obtained using an unmanned aerial vehicle (UAV), and spatial distribution of the leaves along the canopy wall was measured. The vineyard LAI estimation showed a good correlation with R^2^ of 0.82 compared with the traditional method. Indirabai et al. [59] estimated LAI through a ground-based LiDAR scanner and a point spatial density (PSD) algorithm. The algorithm involved filtering and 3D reconstruction of individual trees using super voxel and min-cut segmentation approaches. Experimental results reported a consistent correlation (with R^2^ = 0.96) between the predicted and reference LAI measurements.

### 3.2. Sensor-Based Canopy Measurement Technologies for Precision Spraying

**Figure 3:**
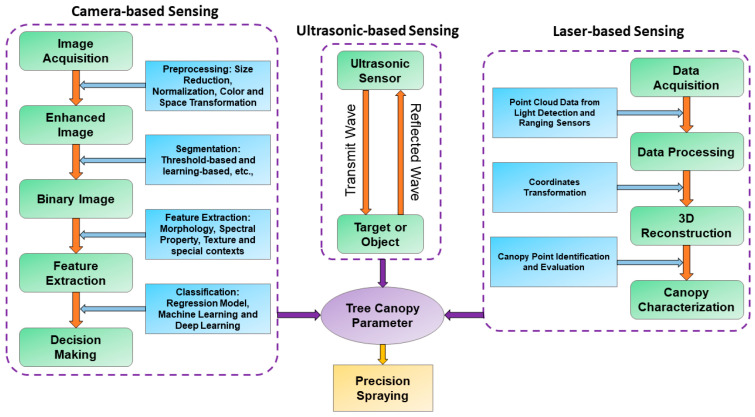
Typical flow-chart for canopy parameter measurement using different sensing systems to support precision spraying.

Measurements of tree canopy parameters require different advanced sensor applications and implementations. The following sections broadly discuss the application of different sensors for precision spraying. Machine vision systems use camera sensors (RGB, NIR, hyperspectral, multispectral, and infrared), and range-sensing systems use ultrasonic and laser sensors to collect tree canopy data and carefully evaluate canopy parameters and targets. Zhang et al. [60] surveyed the different types of real-time sensor applications for variable-rate spraying, including infrared, stereo vision, ultrasonic, and laser scanner. They reported laser scanner and stereo vision sensors as the most promising and complementary techniques to target localization and canopy density estimation of the trees. A general workflow of the target detection procedure using different sensing techniques is illustrated (Figure 3). Three different sensing techniques of camera, ultrasonic, and laser are discussed in this study because these are the main technologies used in precision spraying applications for tree fruit orchards. 

#### 3.2.1. Camera Sensor-Based Technologies for Precision Spraying

Camera-based sensing tries to mimic human perception to provide information or inputs based on processed image data to spraying systems needed for canopy characterization-based pesticide applications. Image processing includes pre-processing, segmentation, feature extraction, and classification. Image pre-processing eases the segmentation of the targeted region (e.g., diseases, canopy, and stresses) from the crop images. It involves image rotation, normalization, resizing, color transformation, denoising and contrast enhancement, etc. Of these, color transformation is one of the most important for segmentation. A detailed description, including the advantages and disadvantages of different color spaces used in machine vision systems, is discussed by Cheng et al. [61]. The conversion of different color spaces used in target detection including channels and transformation function from RGB was thoroughly presented by García–Mateos et al. [62].

After image pre-processing, target area segmentation was carried out. With appropriate target area segmentation different types of targets (e.g., diseased area, canopy, background, gaps between canopies, and so on) can be recognized for precision spraying applications. Various features are being used to differentiate target regions in crops using machine vision applications. Generally, these features are grouped into four sub-classes: morphology, visual texture, spectral, and spatial contexts [63,64,65]. Among them, morphological and visual texture features are most popular and widely applied in orchards for target detections. The extracted features will help the classifier know about the characteristics of different symptoms from the captured images. LWA information and parameters, including distance, area, shape, height, and density, were measured by Xiao et al. [66] using a Microsoft Kinect system. The system consisted of an RGB camera along with a monochrome complementary metal-oxide-semiconductor (CMOS) video camera and an IR transmitter. The Otsu’s method was used to segment the acquired color images to obtain binarized and segmented images from peach and apricot trees. They reported no significant difference existed between LWA of peach and apricot trees of the same size and shape. In the recent study, a novel color-depth fusion segmentation method (CDFS) for collecting the LWA information was proposed [67].

After valuable feature extraction, features are combined to improve robustness when training classifiers/models, which results in high dimensionality of features and the curse of dimensionality. A screening and elimination of unnecessary features is essential to reduce feature quantity and increase data processing speed. Different types of algorithms have been utilized to select effective features for orchard management, including stepwise discriminant analysis [68], quadratic discriminant analysis [69], linear discriminant analysis [70], hybrid artificial neural networks-cultural algorithm (ANN-CA) [71], particle swarm optimization (PSO) based differential evolution method [72], kernel principal component analysis (KPCA) [73], and so on. Upon locating/accessing the target to be sprayed, spray volume at the nozzle is adjusted based on the size or quantity of the target present in the images captured from the orchards.

Deep learning (DL), an artificial intelligence approach, is a new and emerging area of machine learning that offers substantial potential for extracting complex structural information [74] and has seen considerable use in agriculture in the last few years [75]. Compared to the conventional image processing technique, DL algorithms can learn the features themselves from raw data, forming a hierarchy of higher-level features, while the features need to be selectively extracted for conventional machine vision algorithms [76]. Another major advantage is that complex problems can be accurately solved with less time due to the more complex models being used that allow for massive parallelization [77]. The use of DL has been proven for disease detection, fruit recognition, and weed classification, but has not been conducted for tree canopy characterization including the 3D-canopy reconstruction, canopy density, and tree row volume measurements that are needed for efficient tree fruit orchard spraying (to the best of authors knowledge). A few studies conducted in the laboratory have used DL algorithms for precision spraying in weed management [78], but very few DL algorithms have been used for spraying in tree fruit orchards. Seol et al. [79] segmented leaf area for variable flow rate control using four semantic segmentation-based deep learning algorithms including U-Net, SegNet, ICNet, and DeepLab v3 and RGB-D cameras. The SegNet model outperformed three other models and achieved 83.79% canopy segmentation accuracy. Chen et al. [80] conducted site-specific pesticide spraying though Tiny-YOLOv3 deep learning algorithm using an embedded unmanned aerial vehicle (UAV) system and achieved over 95% of pest control.

A few studies have used stereo camera vision-based systems to measure tree canopy shapes and foliage densities and to detect tree crowns [81,82,83]. Malekabadi et al. [81] computed disparity maps through stereo vision to assess the effect of canopy shapes and densities of cherry artifact trees on the sensor performances. Ni and Burks [82] reconstructed citrus tree using a stereo vision system to measure canopy geometry (tree height, width, volume, and leaf cover). The stereo vision system was also used with an UAV to reconstruct 3D tree crowns using watershed segmentation algorithms and local area correlation coefficients [83]. The majority of the stereo vision systems were used for field crop canopy characterizations such as corn and cotton; however, the evaluation of these types of systems for tree fruits is still limited and require more investigations.

**Camera Sensor-based Spraying:** Application of camera sensors for precision spraying in the tree fruit orchards was initiated when different image processing algorithms were available and easily accessible. A summary of the camera sensor applications for variable-rate spraying in orchards is listed in Table 3. Evaluation of camera sensors could be dated back to 1980s but became more popular in 2000s, especially in agricultural applications. At the early stage, a visible range, e.g., red-green-blue (RGB), camera-based machine vision system was used by Thériault et al. [84] to measure the percentage of area covered by tree canopies with a goal of increasing spray recovery in citrus orchards. Results reported spray recovery in the citrus grove ranged from 20% to 45% depending on the size of trees and wind directions. The recovery reached about 61% of the total volume sprayed when the sprayer was tested in a stationary condition without any tree. Hočevar et al. [85] measured the apple tree shape to develop a decision support precision spraying system using an RGB camera and appropriate image analysis. Spraying was performed at a forward speed of 5.30 km·h^−1^ using a sprayer developed by modification-upgrading of a trailed air-assisted sprayer (Agromehanika Kranj, Kranj, Slovenia), equipped with a piston pump and a 1000-liter tank. A total of 23% pesticide saving was calculated compared to spraying in the control mode. Savings were presented in this study for an average tree height of 3 m; however, the saving were not consistent for different fruit varieties with trees of varying shape and size. Suggestions were made that changing the camera position and estimating tree leaf density by scanning the trees may lead to better performance for the orchards with inconsistent tree sizes. Only color information of images was used in their study. A few years later, Xiao et al. [66] captured both color and depth information to adjust and control the spray intensity of sprayers and the dose of sprayed pesticides in orchards. Peach and apricot trees had an average height of 2.5 m, a plant spacing of 3 m, and a row spacing of approximately 4 m. The study revealed the proposed technique could be a key pathway to control the spray intensity of nozzles and the dose of pesticides sprayed, but it was not tested in fields for real-time spraying.

A real-time site-specific orchard sprayer was developed by Asaei et al. [15] to save pesticides in olive orchards based on the tree green canopy detection using a camera vision technology. The experiments showed that the percent of sprayed liquid coverage ranged from 64.62% to 86.42%, 38.52% to 62.50%, and 12.17% to 32.85% for 2, 3.5, and 5 km·h^−1^, respectively, at different positions of the tree canopies. The difference of spray coverage was reported due to the variation of the spatial lag (e.g., the resultant of timing delay and forward speed of the sprayer) during spraying. The study concluded that the spatial lag is influenced by the forward speed could result poor spray deposition.

Aside from tree canopy characteristics detection for spraying operation, few attempts have been investigated based on disease detection. Disease control in grapevines using a totally automatic selective spraying system was first introduced by Oberti et al. [86]. The system consisted of a six degrees of freedom CROPs manipulator, a disease detection system and an end-effector for spraying operation. Three spectral channels were collected including green (540 nm), red (660 nm), and NIR (800 nm) for powdery mildew disease detection. Even though significant pesticide reductions were reported, the acceptance of this technique in real-world applications requires further improvements, particularly in enhancing the ability to measure the latent infection areas around the detected symptoms. On the other hand, Berenstein et al. [87] detected grape clusters and foliage to support precision spraying and reported this can reduce 30% of pesticide usages. The edge detection-based image processing approach was applied for filtering grape green pixels, which is not suitable in field condition because the mask size of the grape is not uniform within the orchards will increase false positive of detection. A few attempts reported in the current year have shown promising potential for pesticide savings by using deep learning-based variable rate spraying [79,80], but more research studies need to be conducted to evaluate its potential more extensively.

For camera-based canopy parameter measurements, the light illumination is the process of making tree canopy clearer or brighter for capturing or acquiring images. Direct sunlight is used as a main source of light in most of the cases for field applications, and under such environments shading and high contrast transitions of illumination are a major problem. The illumination in the field usually varies with different times of a day, the position of the sun concerning the object and overcast conditions which can greatly affect image quality/characteristics [88]. For example, light illumination is comparatively less in early morning and evening as compared to late morning, noon, and afternoon. Bright illumination generally saturates the dynamic range of the camera, which can easily deteriorate the color information of the tree canopy images. Consequently, when the illumination decreases (e.g., low) especially in the early morning, evening, or cloud days, an integral part of the object (tree canopy) surface showing is shaded and occluded which creates significant differences in the diffuse part of bright illumination. The dynamic visible range of a camera is guided by a technological upper limit that results in a sensed image not suitable for processing when the object is irradiated by direct sunlight. Even though the ability of the camera and lens has been improving to compensate for the illumination variations, it is still a major challenge to in-field applications [89]. Apart from light illumination, wind speed [90] and direction, ground speed [88], canopy overlap, and distance between sensor and object [91], etc. are also performance-limiting factors in machine vision applications that need to be addressed.

#### 3.2.2. Range-Sensing Technologies for Precision Spraying

##### Ultrasonic Sensor-Based Technologies

**Figure 4:**
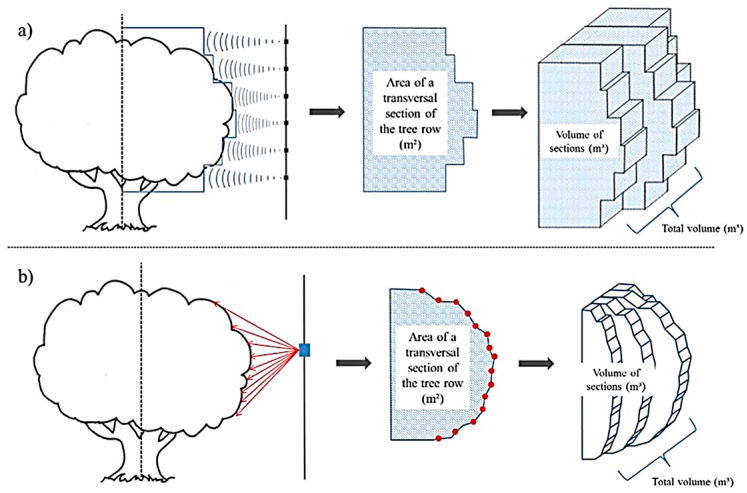
Measurement of canopy volume by using an ultrasonic sensor (**a**) and a laser sensor (**b**) [96] (CC BY 4.0).

Ultrasonic sensor systems are another sensing technology being used to detect the tree canopy parameter to support precision spraying. These types of sensors are affordable, robust, and handy tools that can be used to map target information during spraying [92,93]. To detect the object, sensors transmit waves towards the targets and receive the reflected waves back from them (Figure 3). The ultrasonic sensors can detect the presence of the targets and can measure the distance to the targets based on the time difference between the sound wave being sent and reflected wave being received. The properties and structures of the target are captured by assessing the strength of the reflection [94]. The ultrasonic-sensing system includes an ultrasonic transceiver, an output signal generator, and an amplifier. Various types of transceivers have been utilized and proved to be successful in agricultural applications, including Prowave 400EP250 [95] and Prowave 400EP14D [14]. Stajnko et al. [95] reported a selective amplifier with a 40-dB gain in the frequency of 40 kHz was effective for target spraying in the orchard. Canopy volume and target distance are mainly measured by this sensing technique (Figure 4a).

Ultrasonic sensors for fruit tree volume measurement have been studied widely [97,98,99]. An ultrasonic system (Durand-Wayland Inc, LaGrange, GA, USA) consisting of 20 ultrasonic transducers and ranging boards (10 per side) was used to measure the canopy volume of fifteen trees (heights: 1.7 to 3.3 m) [97]. The estimated tree canopy volumes reported a good correlation of 0.90 with RMSE of 1.66 m^3^ compared to the manual measurements. Experimenting with the same technology, ultrasonic sensors were utilized for tree volume measurement in citrus groves [93]. Various aged trees with different row spacings and groves were measured and obtained a high correlation ranging from 0.95 to 0.99 when compared with manual measurements and ultrasonic measurements. They also found that the effect of forward speed was minor in dense canopy trees compared to the light canopy trees, which was mainly attributed to the more uniform height, diameter, and volume in the dense canopy trees, as well as more openings in the light canopies which weakened the signal echo of the ultrasonic sensor and adversely affected the measurements [98]. The same ultrasonic system was also tested on grapefruit trees with 10 transducers mounted at 0.6-m increments on a 0.1-m diameter vertical PVC mast for tree canopy volume and height measurements [100]. No significant difference was found between manual and ultrasonic measurement where R^2^ of 0.94 (RMSE = 0.16 m^−3^·tree^−1^) and 0.94 (RMSE = 3.12 m^−3^·tree^−1^) were reported for height (2.1 to 4.3 m) and tree volume (6.3 to 54.0 m^3^·tree^−1^) measurements. Ultrasonic-sensing system was used with a differential global positioning system (DGPS) to achieve a variable-rate nitrogen application by generating a prescription map for citrus tree orchards [99]. Results showed the developed system reduced 38% to 40% of fertilizer usages as compared to the uniform application of 270 kg N/ha/y. Palleja and Landers [101] used a variety of ultrasonic sensors to evaluate canopy density in apple orchards with the goal of improving deposition and avoiding drift. The signal obtained from sensors was highly correlated with an error of 3.8% in apple trees; however, the study was not continued for the entire growing season because of increasing apple tree size and the desire to evaluate the performance of the sensor in a specific growing stage (i.e., young apple trees, BBCH: 75). The increase of variability in tree size and shape reduces the accuracy of the ultrasonic sensors when measuring canopy geometric information [102].

**Ultrasonic Sensor-based Spraying:** These types of sensors regulate the spray operation based on canopy parameters measured through sound waves. A summary of ultrasonic sensor applications for site-specific spraying is presented in Table 4. At the initial phase, the performance of ultrasonic sensors was tested in orchard spraying by Giles et al. [103]. The developed system adjusted the flow rate of the sprayer based on the canopy size variations provided by the ultrasonic sensors. Spray savings ranged from 28% to 35% and 36% to 52% in peaches and apples, respectively. 

Balsari and Tamagnone [104] undertook an approach that was able to switch individual nozzles on and off using an ultrasonic-sensing system mounted on a ducted air-assisted sprayer. The sensors were placed at different tree heights to measure canopy volume and showed an average chemical saving of 58%. Using a similar approach, Moltó et al. [105] developed a prototype able to control two electro-valves to apply chemical in two different flow rates (e.g., turn off at the gaps between trees and adjust the valve flow rates according to tree canopy volume) and showed the system was able to save 30% of chemical use in citrus trees. Saving was increased up to 37% when tested in both citrus and olive trees [106].

A prototype spraying system was tested in olive, pear, and apple orchards with an electronic control system equipped with ultrasonic sensors [107], and reported chemical savings from 28% to 70% compared to a conventional spraying technique. Gil et al. [108] stated an average of 58% chemical savings could be achieved using an ultrasonic-sensing system that adjusted to variations of grape structure for spray decisions, as compared to the conventional uniform application. A few years later Llorens et al. [109] achieved mean saving of 58% by applying the ultrasonic-sensing technology in vineyards while also achieving good leaf spray deposition. The nozzles were adjusted according to the variability of tree canopy dimensions.

Maghsoudi et al. [110] utilized three ultrasonic ranging sensors to measure the distance to the target at three different heights to direct precision spray in pistachio orchards. Canopy volume estimation of tree sections was used to make spray decisions. The study reduced the overall agrochemical usage of about 41.3%, 25.6%, and 36.5%, respectively, for the top, middle, and bottom sections of the tree canopy. The magnitude of agrochemical savings was lower due to a higher level of structural variations (e.g., changes of tree width, height, and canopy density) present in pistachio trees. Escolà et al. [102] assessed the ultrasonic sensor performance by measuring the distance to apple tree canopies under laboratory and field conditions. An average error of ±0.53 cm was measured in laboratory conditions, and the accuracy declined with the increase of variability in field conditions. The error of measurement increased when the distance between sensors and target increased, which was due to apple canopies having small surfaces that are difficult to measure at longer distances. Shorter distances between the canopy and the transducer face can provide a stronger returning echo. Additionally, the reflection of sound waves emitted by the ultrasonic sensor is greatly affected by the objects of the measured plane and the directional angle [66].

Different trees have different canopy architectures and leaf angles, and the leaves angle could easily change with the wind [14]. Zaman and Salyani [93] showed that canopy foliage density has a significant effect on ultrasonic sensor measurements of canopy volume. In addition, the performance of the ultrasonic sensors also depends on detection distance and environmental conditions including temperature, and humidity [111,112]. Ultrasonic sensors cannot perform better than laser scanner sensors for estimating canopy volume [97].

The major disadvantage of using an ultrasonic sensor is that the sensor has limited penetration capability, which provides measurements with low spatial resolution. They have limited detection range, and can be greatly affected by canopy detection distance, travel speed, and outdoor weather conditions, which can limit the accuracy of the canopy parameter measurements. Temperature fluctuation affects the speed of an ultrasonic sensor’s sound waves. Sound waves travel faster to and from tree canopies with temperature increases, which results tree canopies appearing closer than they really are. In addition, due to not having scanning capabilities, the ultrasonic sensors provide significantly fewer data compared to other sensing techniques including camera and LiDAR-based sensing, making the ultrasonic technology outdated in precision agriculture as more advanced technologies are already available [96].

##### Laser Sensor-Based Technologies

Laser sensing is an evolving technology mainly used in remote-sensing applications and plant phenotyping that has shown potential in tree canopy parameter detection (e.g., canopy volume and distance of the target) in the last few decades. Compared to other sensors, the major advantage of using a laser sensor for canopy volume estimation is that its electromagnetic signal can penetrate the vegetation canopy to provide canopy density information and the inner and outer structures of the tree [19]. The laser beams transmit visible laser light through a lens towards the object. The laser light is reflected diffusely from the outside of the object and a recipient point of convergence on the sensor focuses that reflected light, making a spot of light on the direct imager. The laser bars receive a variable number of recognized points for each yield from the object cut as indicated by the distance to the sensor and the angle from the horizontal. The basic flow-chart is presented in Figure 3. The sensing process begins with collecting point cloud data using a laser sensor. The raw data accounts for the ground information as well, which must be removed by setting a region of interest. Tree canopy points need to be sorted by removing unnecessary points coming from unwanted regions. The methodology of using a laser sensor for canopy volume measurement is well described by [55,113]. A schematic of the laser-sensing system for canopy characterization is presented in Figure 4b.

Laser scanner sensors can acquire the information and specifications of tree and fruit more precisely than ultrasonic sensors [114,115,116]. Studies reported promising results using laser scanner sensors with canopy volume measurements close to manual measurements. Rosell et al. [117] used a laser scanner system to obtain three-dimensional (3D) structural characteristics of trees in the orchard. The study achieved an accuracy of ±15 mm in a single-shot measurement using a 3D LiDAR sensor with a maximum scanning angle of 180° and a 5-mm standard deviation in a range of up to 8 m. In addition, the correlation coefficient was as high as 0.976 between manually measured volumes and those obtained from the 3D LiDAR models. Another recent study showed that a 3D LiDAR-sensing system could segment the tree canopy, trunk, and trellis system for a trellised high-density apple orchard [118]. The generated canopy density and depth maps could be used in orchard operations such as precision spraying and mechanical pruning. Grau et al. [119] calculated 3D vegetation density using 3D terrestrial LiDAR, and reported the good R2 of 0.91 with RMSE of 20% for a surface density up to 2 m^2^·m^−3^. The study revealed the error increased with angular resolution of LiDAR and vegetation density. Li et al. [120] used the high-resolution terrestrial LiDAR to measure leaf area density of individual trees and reported lower accuracies of 86.53% and 84.63% for two trees due to the application of inappropriate voxel size. Hosoi and Omasa [121] also measured tree leaf area density using high-resolution LiDAR and achieved errors of 17% due to high obstruction of laser beams. The study reported the inclination of the laser beams could provide less obstruction and better penetration of laser beams into the canopies, yielding better leaf density measurements. Rosell Polo et al. [115] utilized a 2D LiDAR to measure tree row volume and the total crop surface area. A strong R^2^ of 0.9738 was reported between the LiDAR measured plant volume and the tree volume measured manually. Therefore, the above studies concluded that the LiDAR-sensing system could be a powerful technique for non-destructive estimates of canopy foliage area characteristics and canopy density measurements of trees. LiDAR-sensing technologies are also widely utilized for determining spray drift and buffer zones in 3D crops [122] showing that LiDAR sensor could be able to determine the buffer zone in grape, citrus, peach, and apple orchards.

**Laser Sensor-based Spraying:** In the last decade, LiDAR-based laser-sensing technologies have been tested in fruit orchards due to their high precision and accuracy in real-time measurements [12,123]. A summary of laser sensor applications for site-specific spraying is presented in Table 5. Although laser-sensing technology was extensively investigated for sprayer development before the 2010s, it was first integrated successfully into a precision sprayer by Chen et al. [124]. This system measured tree canopy structure and adjusted control nozzles based on the height and width of the target trees. There were acceptable variations in coverage inside the tree canopies, with coefficient of variations (CV) ranging from 52% to 79%, 27% to 53%, 11% to 33% in the X, Y, and Z directions, respectively. However, the main restriction of this study was that spray volume, spray coverage, and uniformity inside canopies diminished as the movement speed increased from 3.2 to 6.4 km·h^−1^, because the necessary spray output surpassed the nozzle limit at a higher speed. The authors concluded that the measurement of different canopy foliage structures and densities need to be accounted for when evaluating the variable rate sprayer performance. 

Another major limitation in using a laser sensor in this attempt was that it required two sensors for real-time detection of trees on both sides of the tree row due to the maximum radial scanning range of 180° in the laboratory-based evaluation. Considering the scanning limitations, Liu and Zhu [12] measured the dimensions of target surfaces with complex shapes and sizes using a 270° radial range laser sensor to develop a variable rate sprayer. The surface/boundary of the trees and distance between the sensor and object were measured. The performance was better during laboratory-scale tests with artificial trees compared to field-grown ornamental trees. However, the study did not consider the canopy volume of the trees, which is very important to accurately measure the tree architecture, with this being a major limitation of this study. 

Using the same sensor configuration as Chen et al. [124], apple tree canopy characteristics including height, width, volume, foliage density, and occurrence were measured to control nozzles for real-time orchard spraying [49,125]. Three different phenological stages (e.g., leafing, half-foliage, and full-foliage) were tested. Among the three growth stages, the precision sprayer provided more consistent results on spray coverage and deposition during the leafing stage compared to the half-foliage and full-foliage stages. A few years later, Chen et al. [125] utilized the same laser-guided variable-rate spraying system for controlling insects and diseases in ornamental nurseries. The developed system provided equivalent or greater control of insect and disease as compared to the conventional sprayer.

Boatwright et al. [16] compared a LiDAR-based variable rate sprayer system with a conventional spraying approach for disease and pest control management in a peach orchard. They reported the LiDAR-based precision sprayer could have the same amount of control with chemical savings ranging from 13% to 50% at various phenological stages (i.e., bloom, pit hardening, and final swell) of the trees. The spray coverage was increased at the bloom (50.13%) and pit hardening (26.67%) stages, but no improvement was noticed in the final swell stage.

In the recent study, Chen et al. [126] conducted a comparative analysis between intelligent sprayers using a laser sensor and constant-rate conventional sprayer for chemical usage and pest control. The pest severity of the apple trees were rated, including codling moth, powdery mildew, scab, and oriental fruit moth. This study reported similar findings, where the pest control performance was also equivalent to or greater than the conventional method.

Manandhar et al. [127] conducted an economic analysis between laser-guided variable-rate sprayers and conventional constant-rate sprayers for spraying in the apple orchards. They concluded a potential pesticide cost savings between $1420 and $1750 ha^−1^ is possible along with reductions of spraying time by 27–32% and labor and fuel by 28%.

Apart from the chemical saving, Shalal et al. [123] detected apple tree trunks using a laser scanner sensor and achieved 96.64% of detection accuracy. The study revealed the laser scanner sensors are capable of providing reliable angles and width of tree trunks and information on nontree objects. Overall, the results of the above studies were satisfactory, but the extrapolation of these results to trees with different structures is not easy.

Challenges are also associated with laser sensing, especially when vibrations from terrain variability are presented during scanning for tree canopy data. Inconsistencies of terrain could cause deviations of the angular orientation of the LiDAR sensor during examining/scanning [112], which could be corrected using a positioning sensor such as inertial measurement unit (IMU) sensor. These types of sensors acquire huge datasets during scanning of the trees that require a high level of analysis and interpretation. They do not work well in areas where there are high sun angles or huge reflections due to the dependency of the principle of reflection. In addition, the LiDAR pulses may not be able to penetrate through dense canopies, especially in forests, and can provide incomplete data, which should be considered for future sprayer development.

### 3.3. Conclusions for Sensor-Based Precision Sprayers

Estimating canopy size is a challenge because of the confounding structures and irregular shapes of trees, and variable environmental conditions in fields. Problems associated with camera vision sensors in field conditions include variable light, and distorted or blurred images due to vibrations and wind direction and velocity [15], which can easily influence the performances of the system. Consequently, the major drawback of ultrasonic sensors is the large angle of divergence of ultrasonic waves that also needs to be calibrated. In addition, very uneven fields generate inconsistent data [60]. Apart from vision and ultrasonic sensors, the spectral and infrared sensors are also not feasible to detect tree canopy structure information in tree fruit orchards due to being highly sensitive to outdoor illumination variations and environmental conditions [60], and are not commonly used in precision spraying. Comparative to other sensing systems, a laser scanner sensor could be a viable option to measure canopy structure characteristics aimed at precision spraying in the orchards. Environmental conditions do not influence laser sensors and can provide more accurate detection of tree canopy structure [117,125]. The advantages and disadvantages of different sensing systems used in agriculture for variable-rate spraying are summarized in Table 6.

Although several groups of researchers have developed prototypes to change the application flow rate based on canopy structural parameters from different sensors, precision spraying in tree fruit orchards needs further development to deal with excessive chemical usages, improper spray depositions, and losses from chemical drift [16]. Real-time precision orchard sprayer development is a challenging endeavor due to outside, uncontrollable environmental conditions. A laser sensor could be a solution to the problems associated with accurate canopy structure information measurement. Still, the temperature, humidity, wind speed and direction, and land topography have direct effects upon spray deposition to the target that needs to be accounted for. For example, high temperature and lower humidity can cause faster evaporation of the spray drift before reaching the target.

## 4. Other Factors Affecting Performance in Spraying

The effectiveness and quality of spraying depends on many factors, including spray deposition, drift, leaf absorption, and coverage volume. Spray penetration and coverage are greatly influenced by tree height, tree spread, and the continuity of canopies along rows which may easily reduce the effectiveness of the crop protection operation [132]. In this section, we discuss additional factors that affect spraying performance.

### 4.1. Sprayer Specifications

#### 4.1.1. Travel Speed

Several studies have analyzed the effect of forward speed on spray deposition and drift [93,133]. Salyani [134] reported an inverse relationship between deposition and travel speed, and deposit uniformity and travel speed. Others reported a non-significant effect of travel speed on spray deposition for variable-rate sprayers using ultrasonic sensors for canopy detection [93,111,135], although the uniformity of deposition decreased as the speed increased due to bent and distortion of air-jet [133]. Whitney et al. [136] reported an enhanced deposition on the upper leaf surface with increasing forward speed of air blast sprayers in citrus, but no significant increase in the deposition was reported on the lower leaf surface. The results did not correspond to other studies, which may be due to the depth of the sample collection as the higher canopy density results in less spray deposition. As the distance from the sprayer increases, the amount of deposition and uniformity decreases. The spray penetration into the canopy is influenced by the interaction of wind velocity and sprayer ground speed as it affects the air jet generated by the sprayer [135] and reduces the spray penetration and uniformity with increasing travel speed. However, all these reported studies indicated that both the penetration and uniformity are low at the higher travel speed; therefore, the travel speed should be considered for applications where the spray uniformity and canopy depth is critical.

#### 4.1.2. Distance

The distance between sprayer and canopy is another important parameter that influences the amount of spray drift [137]. Celen et al. [138] reported that increasing the travel speed and sprayer-canopy distance increases the spray drift, causing less deposition on the target. The droplet size of chemicals is important in understanding potential spray drift. Although smaller droplets can potentially enhance canopy coverage, they are more prone to drift to non-target areas [138]. Derksen and Gray [139] reported the influence of the distance between the spray outlet and canopy on spray deposition and drift using air blast sprayers. Increasing the distance between the sprayer and the target canopy resulted in decreased deposition levels in trees. Wandkar et al. [133] reported that deposition on guava trees was significantly affected by canopy depths at different travel speeds. This was due to the position of the canopies of the trees, with maximum spray deposited on the upper leaf surface. Keeping the nozzles close to vegetation can reduce the potential of spray drift without affecting the uniformity of the coverage and spray pattern [140].

#### 4.1.3. Mechanical Fan and Air-Flow Control

The mechanical system performing the spraying operation also affects the spray deposition and off-target losses in orchard trees. Salyani [134] reported an increase in spray deposition on targets in an open area using an axial fan air blast sprayer at a higher travel speed. Steinke et al. [141] used tower air blast sprayers to compare the effect of different fans on spray deposits and reported a more uniform deposition using crossflow fans than a conventional axial fan. Similarly, using an air blast sprayer with horizontal delivery resulted in increased spray deposition and low off-target drift compared to the axial flow air system [142]. Pergher and Gubiani [143] reported a high spray deposition (54–57%) in vineyards with a combination of lower air flow rate and lower spray volume. Wandkar et al. [133] reported that spray deposition increases with increasing air jet velocity in the dense canopy, while Van de Zande et al. [144] reported higher losses to deposition on the ground with air-assisted sprayers as compared to conventional boom sprayers.

#### 4.1.4. Nozzles

The spray drift is also influenced by the type, size, and pressure of the nozzles used for spraying. Spray nozzles should be selected by following the required spray volume [143], nozzle type, and pressure [145] to improve the coverage and penetration into the canopy. Wandkar et al. [133] compared two different spray nozzles (hollow cone and flat fan nozzles) and concluded that the nozzle with smaller droplet size (hollow cone nozzle) shows higher deposition. Studies conducted by Zhu et al. [146] reported a higher spray deposition on the lower part of the canopy due to a greater percentage of large size droplets produced by the air-assisted nozzle as compared to hollow-cone nozzles, while [145] conducted a field study using a crossflow orchard sprayer and reported the drift reduction from air-inclusion nozzles compared to hollow-cone nozzles is significant beyond 8 meters from the last row of the orchard. Considering the effects of nozzles in spray drift losses, a nozzle classification system was developed to identify the drift reduction potential of spray nozzles used in fruit crop spraying [147]. The study reported the highest drift reduction of 97% could be obtained with a nozzle (Albuz TVI 80025) based on V100 (volume of drops smaller than 100 μm) when sprayed at 7-bar spray pressure. The suggested threshold nozzles for these drift reduction classes in orchard spraying are, respectively: TeeJet DG8002 (drift reduction of 62%), Albuz AVI 80015 (drift reduction of 74%), and Albuz TVI 80025 (drift reduction of 97%), all at 7-bar spray pressure, as well as Lechler ID 9001 (drift reduction of 91%) at 5-bar pressure. Another study revealed that the V100 achieved a high correlation (up to R^2^ = 0.948) with the drift potential tested with the wind tunnel for hollow-cone nozzles [148].

### 4.2. Meteorological Condition Effects

#### 4.2.1. Wind Speed and Direction

**Figure 5:**
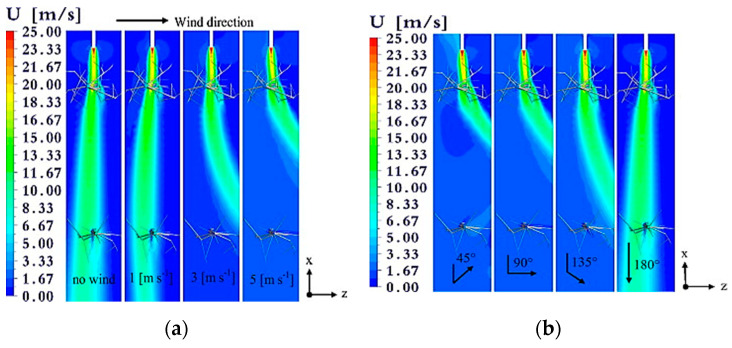
Effect of wind speed (**a**) and direction (**b**) in spraying using CFD analysis [149] (used with permission).

Among the atmospheric factors, the wind speed and direction have tremendous effects on the spray depositions causing off-target agrochemical losses to the non-target receptors, including damage to the neighboring crops, water, and animals. The rate of off-target losses depends on the speed of the wind and direction of the wind flow (Figure 5).

In general, all types (class-based on sizes) of droplets have the chance of being off-target depositions, but the small droplets have the highest possibility of the off-target movement. Endalew et al. [149] utilized a computational fluid dynamics (CFD) based simulation model to evaluate the effect of wind speed and direction on sprayer airflow. A reduction of 2 m·s^−1^ airflow was reported from a two-fan air-assisted sprayer when there was a crosswind speed of 5 m·s^−1^ with the direction of 90° that could significantly affect spray deposition. In addition, a new model of bystander and resident exposure was developed to measure spray drift deposition from orchard applications [150]. Observations were made in three growth stages (dormant (BBCH 0‒60), intermediate (BBCH 61‒73 and 93‒0), and full leaf (BBCH 74‒92)) of the apple trees. The spray drift depositions on soil surface were 11%, 15%, and 23% for the full leaf, intermediate, and dormant stages, respectively, while spraying from 5-m apart using a crossflow fan sprayer. The functions and its parameters for the three growth stages are: dormant: Y = 38.797 × 10^−0.104X^; intermediate: Y = 26.928 × 10^−0.124X^; full leaf: Y = 19.036 × 10^−0.118X^; where Y is spray drift deposition (% of sprayed volume) at distance X m from the last tree row.

#### 4.2.2. Temperature

Although the effects of temperature on spraying are region-specific (e.g., mainly occur in temperate/hot region), there is a slight deviation of the spray drift potential observed by the researchers due to evaporation [151,152]. Water is the most used liquid for mixing with agrochemicals to be applied in crop protection applications. Due to the characteristics of water, the surface tension of water decreases significantly with increasing temperature, which results in the decreasing size of the spray droplet. Holterman [151] reported that high air temperature could cause evaporation of spray drift and make them drift-prone by lessening the droplet size using a flat fan nozzle. Downer et al. [153] made a similar conclusion where a decrease of the spray droplet size with increasing temperature was also reported, and, meanwhile, the authors observed the surface tension of all spray liquids was also decreasing. To access the effect of temperature, Miller and Tuck [154] used a flat fan and an air induction nozzle and noticed the temperature had a direct effect upon the spray droplet size. Despite the temperature effect being slight compared to the other factors, the effect could be significantly impacted by hot regions, especially in the summer months.

#### 4.2.3. Relative Humidity

**Figure 6:**
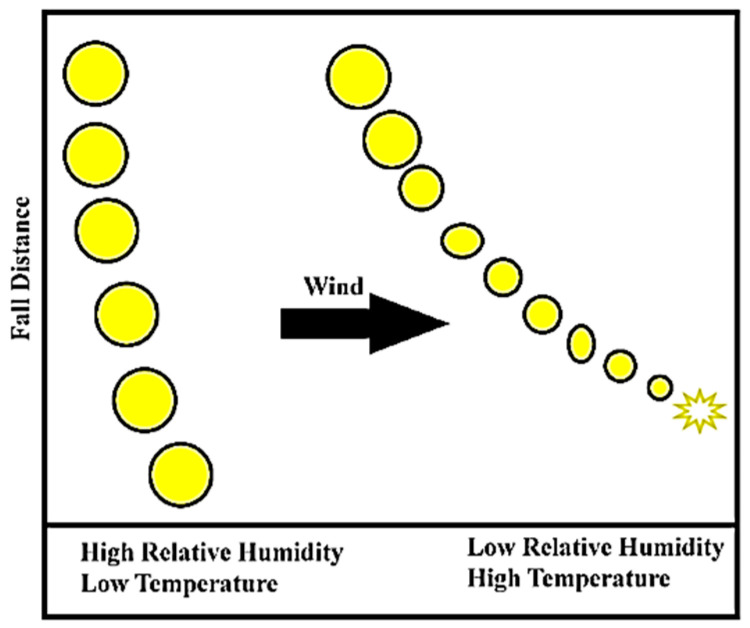
Effect of humidity and temperature on spray droplet size and movement.

Humidity is another region-specific factor that affects parameters by decreasing spray droplet size, especially in the lower humidity regions [151]. Humidity, along with high air temperature, affects the evaporation of liquid droplets [151,155]. Hanna [155] reported the evaporation rate of spray droplets is comparatively faster when the humidity level is low, and air temperature is high. The reduced droplets are more susceptible to off-target movement with prevailing winds as they become entrapped in ambient air currents [155] (Figure 6). Studies conducted to compute the ideal condition for spraying considering different meteorological conditions. An absolute humidity of 8 g·kg^−1^ along with a 16 °C air temperature and a wind speed of 3 m·s^−1^ are considered as ideal meteorological conditions [152].

### 4.3. Interaction of Pesticides and Leaves

#### 4.3.1. Droplet Size

Several factors influence spray coverage, including droplet size, carrier rate, and formulation [137]. A different group of researchers worked in laboratories to demonstrate the effect of droplet size on the spray’s biological efficacy and reported improved insect control from using smaller droplet sizes [156,157]. Salyani and Cromwell [7] reported reduced spray drift in a laboratory study using drift retardants, and further studies agreed as the median diameter of spray increases using drift retardants [158]. However, extensive care and time should be considered to properly blend drift retardants with spray carriers [159].

#### 4.3.2. Retention

Retention of spray droplets on leaf surfaces depends both on the characteristics of the surface and of the liquid and is critical for effective spraying operation. Spray retention is significantly reduced when the velocity of spray droplets is higher due to high nozzle pressure [160]. The droplet also tends to bounce when applied at higher pressure and velocity. The retention of spray deposits on the surface of horizontal and vertical canopy surfaces is crucial. Feng et al. [161] reported retention efficiency with the application of fine, coarse, and medium-size droplets as 47%, 38%, and 37%, respectively. Dorr et al. [160] reported that the droplet size over 400 micrometers is likely to shatter off the surfaces, which explains why high spray deposition was reported at a higher pressure. The authors explained it might be because the velocity is high at higher pleasure, which resulted in high deposition. Spillman [162] noticed the highest deposition efficiency on leaves for droplets size greater than 250 micrometers, while small size droplets (20–50 micrometers) have better deposition efficacy on the vertical surface, including lower leaf surface.

## 5. Discussion and Future Directions

This review has discussed the current methods of spraying in orchards, machine vision applications for precision spraying, implementation of sensor-based precision spraying systems in orchards, and limitations of different sensors and corresponding challenges. The tree fruit industry has gained considerable attention due to its high usage of agrochemicals in orchards. In the last few decades, various type of spraying systems, including handguns, boom sprayers, polar jets, tunnel air blast sprayers, and so on have been tested in orchards to try and reduce chemical usage; however, none of them was fully successful and satisfactory due to non-uniform tree canopy structures and sprayer limitations.

Although various machine vision approaches have proved to be very useful and successful in weed detection, crop stress monitoring and yield prediction, etc., challenges still exist, especially in outdoor field conditions. Among the different types of sensors used in machine vision applications, camera sensors are more susceptible to outdoor lighting and weather conditions. The camera vision approaches might be best suited for spot spraying (spray based on site-specific disease or pest pressure) rather variable-rate spraying in the tree fruits. Recent developments of multispectral and hyperspectral cameras offer narrower spectral bands that can penetrate into the canopies and accurately measure the conditions (diseases or pest pressures and stresses because of nutrients) of trees. However, longer data processing time (particularly for hyperspectral cameras) is a major limitation. Current inventions of deep learning-based artificial intelligence systems resulted in good canopy detections and segmentations. Semantic and instance segmentation based deep learning models, including SegNet, U-Net, DeepLab v3, mask-region-based convolutional neural networks (RCNN), and so on, could be the best fit for canopy area segmentation; however, more efforts and extensive investigations are expected to develop an effective and robust system for precision spraying. Apart from camera sensors, ultrasound and laser scanner sensors can overcome light illumination problems usage sound waves and electromagnetic signals, respectively, as inputs to measure detection distance and gather canopy structure parameters. As compared to ultrasonic and camera sensors, laser scanner sensors are more accurate and reliable in collecting crop structure information, are not affected by weather conditions, and can guide the sprayer unit efficiently and effectively to apply pesticides on target.

The application of laser scanners has risen during the last decade in multiple agriculture applications, from automatic tree height measurement to site-specific pesticide application. However, challenges are still presented, especially in site-specific or variable-rate spraying in tree fruit orchards. Accurate canopy information measurements require automatic adjustment of laser sensor orientation during data acquisition, especially in conditions of different sloping and uneven terrains. Therefore, a real-time terrain monitoring system may need to be incorporated along with a sensing system for precision spraying. Terrain monitoring systems can be developed using a positioning sensor and a real-time kinematic global positioning system (RTK-GPS). An inertial measurement unit (IMU) sensor can be incorporated into the spraying system to monitor the terrain conditions in real-time and adjust the laser orientation accordingly. Studies reported IMU sensors could minimize positioning errors of the laser from terrain variabilities [96,112]. The RTK-GPS will locate the uneven terrain position and guide the spraying system to follow the co-ordinate where the necessary adjustment is needed for accurate canopy information. Few studies have considered uneven terrain information for correcting tree canopy information [163,164], so more studies need to be conducted to implement real-time correction of tree canopies for accurate spray deposition.

**Figure 7:**
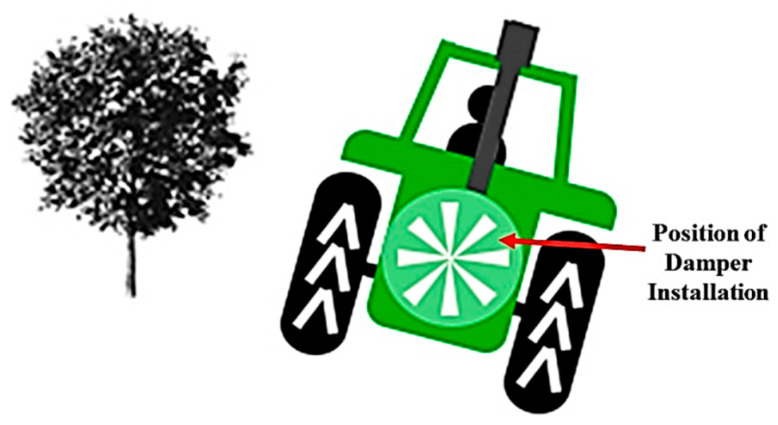
Position of damper installation for precision sprayer airflow control.

Laser scanner sensors only help with measuring canopy structure information, but a successful spraying operation requires a series of tasks to be completed accurately, including maintaining constant travel speed, adjustments of the fan speed at the sprayer unit, assessment of real-time weather conditions, prediction of droplet sizes and some other factors. Auto-guidance systems that automatically steer the sprayer throughout the orchard blocks could help maintain constant travel speeds. Crossflow fan sprayers reduce off-target spray deposition because they have more appropriate nozzle positions and uniform air delivery. An automatic damper system can be added at the sprayer fan’s air inlet (back of the sprayer) to control the airflow based on tree canopy information (Figure 7). A mathematical model will need to be developed to calculate the airflow required for sending the spray droplet to the desired canopies.

Real-time weather assessment is also important to reduce off-target deposition. Wind can easily blow spray from targeted to non-targeted regions. The real-time control of nozzles based on weather data is not currently practical during spray operation. The future sprayer also needs to include a droplet size prediction model to determine the diameter of droplets based on the characteristics of the tree canopies during spray operations. Electrostatic sprayers have provided improved deposition of specific droplet sizes and may be a good aid to develop next-generation sprayer systems [165]. Electrostatic sprayers apply a positive charge to liquids as they pass through the nozzle. The positively charged liquids are attracted to negatively charged surfaces, which allows for efficient coating of hard nonporous surfaces. A 44% increase in mean deposition using the electrostatic system reported by Pascuzzi and Cerruto [166]. Some other factors including canopy conditions and spray droplet retention depend heavily on the tree canopies and cannot always be addressed by the spraying unit. However, the nozzle effect can be resolved by matching appropriate nozzles with electronically controlled valves.

The future of precision spraying systems will focus on integrating a suite of robotic and sensor-guided spraying systems with fruit tree growth and structure information to ensure accurate dose of pesticide delivery and reduce off-target deposition. A wide variety of sensor-based spraying technologies are available to measure the amount of spray volume needed; however, it is also important to assess the durability of these technologies so that a variety of sensors can be added at reasonable price for the farmers. Concurrently with sensor development, site-specific disease, pests, and stress monitoring using technologies such as deep learning and remote sensing will improve the functioning of current sensor-based spraying systems. Fusing/integrating different sensors could help to add more decision-making features for more precise pesticide applications in tree fruits. Future spraying systems must also handle diverse agrochemical formulations beyond that of the standard aqueous mixtures currently used. Although tractor-based spraying systems are mostly used for agrochemical applications, an alternative UAV based application method could displace traditional sprayers. UAVs have been tested in the field crops for disease scouting, stress monitoring, and spraying, and its preliminary results for spraying is showing promise. But most of the current UAV based spraying technologies may not be suitable for spraying in large scale tree fruit orchards due to their limited payloads and variable spray deposition capability. More research and development into UAV swarms and large capability UAV similar to unmanned helicopters could result in more effective system for commercial/large scale fruit orchards. Detection of disease and stress, pest pressure are other avenues for their use in tree fruits production besides variable-rate spraying. Therefore, the integration of multiple sensing and control systems will be the key to using advanced precision sprayers in the future.

## 6. Conclusions

The excessive use of agrochemicals by conventional sprayers has gained attention among orchard growers, practitioners, and ecologists. Serious efforts are required to make an advanced precision orchard spraying system commercially available in an economically and environmentally sound manner. Considering the precision, accuracy, and numerous factors of using different sensors in outdoor environments, we conclude with the following guidelines for variable-rate spraying in the tree fruit orchards:

- The canopy geometry, canopy density, leaf area index, and leaf area density are essential for variable-rate spraying and could be measured accurately with high precision using ultrasonic and LiDAR sensors;
- The camera sensors-based sprayer can be a good aid for detecting position of the canopies and spot spraying; however, their performance is inferior due to environmental and sensor limitations;
- The sprayer with ultrasonic sensors can have high precision in the orchard where the trees are discontinuously planted or trees with low canopy density and less variation among sections. These kinds of sensors can be useful for measuring average target canopy characteristics;
- The LiDAR sensors guided sprayer could be suitable for many types of orchards (low, medium, or high canopy density) and provide canopy details regardless of the environmental conditions with comparatively high reliability and accuracy;
- Use of LiDAR sensors must need advance algorithms and microprocessors to overcome the complex filtering problem and to process large amounts of information;
- Fusion of different advanced sensors, application of new algorithms such as deep learning, and new research methodologies should be considered to develop the next generation advance precision sprayer for tree fruit orchards.

## Figures and Tables

**Table 1:** Revolution of sprayers used in orchard spraying.

Evaluation	Sprayer Types	References
Early Sprayer Design	Steam-powered sprayers; boom sprayers and early mist blowers; handguns	[20,22,23,24]
Air Jet Models	Polar jets; plane jet sprayer	[25,27,28]
Modern Air blast Sprayers	Tower sprayers; tunnel sprayers	[29,30,31]
Precision Sprayers	Sensor guided air blast sprayers; intelligent sprayer	[16,26]

**Table 2:** Crop structure parameter equations for precision spraying in orchards ^1^.

Name	Crop Structure Parameter	References
Crown height	H	[38]
Leaf Wall Area (LWA)	$\mathrm{LWA}=10000\frac{H}{B}$	[39]
Tree Row Volume (TRV)	$\mathrm{TRV}=10000\frac{W\times H}{B}$	[40]
Leaf Area Density (LAD)	LAD ∝ A	[41]
Tree Canopy Density (TCD)	$\mathrm{TCD}=\frac{N_{p}}{T_{A}}$	[42]
Leaf Area Index (LAI)	$\mathrm{LAI}=\frac{A\times W\times H}{B}$	[43]

^1^ Where A in m^2^/m^3^ (leaf area per unit canopy volume) is the leaf area density; B in m is the row spacing; H in m is the height interval of the canopy; W in m is the canopy width; LAI (leaf area per unit ground area) is the leaf area index; $N_{p}$ is the number of pixels or points counted in a targeted region; $T_{A}$ is the area of the targeted region in m^2^.

**Table 3:** Summary of camera sensor used in precision spraying in orchards.

Crops	Sensors	Detected	Accuracy	Chemical Saving	Limitations	References
Orange and grapefruit	RGB camera	Tree canopy	Not reported	A saving of 22% to 45% was reported in the citrus grove	The reported savings could be achieved only for spraying small trees	[84]
Apple	RGB camera	Tree canopy	Not reported	23% of saving of pesticides (0.96 L min^−1^ flow rate reduction)	Size and shape variability of trees was not considered for claiming the amount of saving	[85]
Olive	RGB camera	Tree canopy	Greenness detection was not reported	Savings of up to 54% in pesticide usage compared with conventional continuous spraying	Reduction of pesticides is only considered in the gaps between trees	[15]
Grapevine	Multispectral camera	Powdery mildew disease	Detected about 85% to 100% of the diseased area	A reduction of 65% to 85% was reported based on site-specific spraying to the diseased areas	False-positive of the developed system was from 5% to 20%	[86]
Grape	RGB camera	Clusters and foliage	Over 90% accuracy was achieved for both cluster and foliage detection	Reduction of 30% in the use of pesticides	Algorithm processing time was longer and not appropriate for real-time application	[87]
Pear	Kinect RGB-D camera	Fruit tree	Highest accuracy of 83.79% was reported using SegNet model	Pesticide application was reduced up to 56.80%	Number of trials was insufficient require extensive investigation	[79]
Litchi	Optical camera	Pest detection	Up to 95.33% average precision was achieved	Reduce spray volume by 87.5%	-	[80]

**Table 4:** Summary of ultrasonic sensor used in precision spraying in orchards.

Crops	Detected	Accuracy	Chemical Saving	Limitations	References
Peach and apple	Canopy foliage	Not reported	28% to 35% for peaches and 36% to 52% for apples	Spray deposition was reduced on some canopy areas	[103]
Apple	Tree canopy volume	Not reported	Approximately 58%	The detected vegetation gap width was between 0.35 and 1.20 m. Smaller gaps could not be identified because of the wide-angle field of view of the sensors	[104]
Citrus	Tree canopy	Not reported	The system achieved 30% of saving in time	Handgun sprayer was used for spraying	[105]
Citrus and olive	Tree shape and gap between trees	Not reported	Saving up to 37%	Only considered the gap between trees to support variable-rate spraying	[106]
Olive, pear, and apple	Tree canopy width	Not reported	Savings of 70%, 28%, and 39% were reported for the olive, pear, and apple, respectively	Droplets from the nozzle did not follow a straight trajectory which caused lower spray deposition on the tree canopy	[107]
Grape/vines	Tree row volume	R^2^ = 0.99 for distance between sensor and crop measurement and R^2^ = 0.97 for leaf area determination	Average of 58.8%; savings were 83.9%, 32.7%, and 48.0% at the lower, the top, and middle parts of the crop, respectively	The experiments were conducted at the very late crop stage (BBCH > 80: ripening stage) where a majority of the leaves was large and uniform in size compared to early and middle stages, which caused less variability	[108]
Grape	Tree row volume	R^2^ of 0.66 was reported for TRV measurement	58% of application volume	Experiments did not consider the effects of ground speed	[109]
Apple	Contour of the tree canopy	Not reported	20.2% per nozzle	The savings varied by the size and training of orchard trees	[14]
Apple	Distance measurement of apple tree canopies	Average errors of ±0.53 cm, and ±5.11 cm in laboratory and field scales	Did not spray	Increase of variability in field conditions significantly reduced the accuracy of the sensor	[102]
Pistachio	Volume estimation of tree sections	R^2^ value of 0.99 for training and 0.96 for testing data was reported using artificial neural network (ANN)	34.5% overall, 41.3%, 25.6%, and 36.5%, for top, middle, and bottom canopy sections, respectively	The magnitude of chemical savings was comparatively lower than other studies, especially in the center of the trees	[110]

**Table 5:** Summary of laser sensor used in precision spraying in orchards.

Crops	Detected	Chemical Saving	Limitations	References
Apple and peach	Tree canopy foliage volume	52.4% for apple and 34% for peach	Canopy density characteristics were not considered, resulting in more chemical savings in smaller trees compared to larger and denser trees	[126]
Peach	Tree canopy volume	50%, 40%, and 13% at bloom, pit hardening, and final swell, respectively	Spray coverage was not good at the final swell	[16]
Apple	Tree canopy foliage volume	Two year average of 60.5% by volume	Only trees with small canopies were tested	[128]
Apple	Tree height, width, volume, foliage density	47% to 73% at the growth stages of leafing, half-foliage, and full foliage	Uniform chemical spray coverage and deposition were reported along with the canopy axes of depth, width, and height	[49]
Apple	Tree canopy height, width, and foliage density	Average of 68% to 90% on the ground, 70% to 92% around tree canopies, and 70% to 100% of airborne spray	The results were not consistent, and variations were reported between half-foliage and full-foliage stages	[125]
Apple	Canopy volume	Approximately 46%	Experiments did not consider different growth stages	[129]
Crab-apple	Tree size, shape, and leaf density	Spray coverage on the foliage of trees was 19.86% ± 3.0%	Experiments were only conducted in nursery field conditions	[130]
Maple	Canopy density and tree shape	Spray area coverage was increased by 30% to 55%	Spray coverage was higher on the front side as compared to the backside position	[131]
Artificial and ornamental trees	Shape and size of trees	Did not spray	Performed better with artificial trees than with ornamental trees	[12]
Apple	Tree canopies	Reduced pesticide costs by 60–67%	This study only compared the economics of variable-rate and constant-rate sprayers	[127]

**Table 6:** Advantages and disadvantages of the different types of sensor applications for precision spraying (modified from [60]).

Sensors	Pros	Cons
Camera sensors	Provide information about crop diseases, pests, weeds, and nutrient deficiencies Ability to measure tree canopy area Less expensive	Highly sensitive to the illumination conditions 3D reconstruction of tree canopies is very difficult Weather conditions have significantly affected the sensor performance
Ultrasonic sensors	Robustness and low price Capable of determining tree canopy structure characteristics Relatively easy to implement	Limited resolution and accuracy of the measurements Detection distance and environmental conditions can affect sensing accuracy Require multiple sensors to sense plant structure
LiDAR sensors	Independent of environmental conditions Rich data acquisition capability High speed of measurement Provide high resolution of tree canopy structure characteristics Plant data, for example, height, width, volume, leaf area index, and canopy density, can be acquired with adequate precision	Tractor bouncing can affect data acquisition which requires correction Delicate moving part inside the sensor

## Data Availability

Not applicable.
